# Cost-Effectiveness of Peer-Delivered HIV Self-Tests for MSM in Uganda

**DOI:** 10.3389/fpubh.2021.651325

**Published:** 2021-03-17

**Authors:** Stephen Okoboi, Barbara Castelnuovo, Jean-Pierre Van Geertruyden, Oucul Lazarus, Lung Vu, Sam Kalibala, Yvonne Kamara, Perez N. Ochanda, Rachel King, Andrew Mujugira

**Affiliations:** ^1^College of Health Sciences, Infectious Diseases Institute, Makerere University, Kampala, Uganda; ^2^Faculty of Medicine and Health Sciences and Global Health Institute, University of Antwerp, Antwerp, Belgium; ^3^Global Health Institute, Clarke International University, Kampala, Uganda; ^4^The AIDS Support Organization (TASO), Kampala, Uganda; ^5^Population Council, Washington, DC, United States; ^6^Department of Global Health, University of California, San Francisco, San Francisco, CA, United States; ^7^School of Public Health, Makerere University, Kampala, Uganda

**Keywords:** MSM, HIV, self-testing, peers, cost-effectiveness, Uganda

## Abstract

**Introduction:** Distribution of HIV self-testing (HIVST) kits through MSM peer networks is a novel and effective strategy to increase HIV testing coverage in this high-risk population. No study has evaluated the cost or cost effectiveness of peer distribution of HIVST strategies among MSM in sub-Saharan Africa.

**Methods:** From June to August 2018, we conducted a pilot study of secondary MSM peer HIVST kit distribution at The AIDS Support Organization at Entebbe and Masaka. We used an ingredients approach to estimate the cost of MSM peer HIVST kit distribution relative to standard-of-care (SOC) hotspot testing using programme expenditure data reported in US dollars. The provider perspective was used to estimate incremental cost-effective ratios per HIV infection averted using the difference in HIV annual transmission rates between MSM with HIV who knew their status and were not virologically suppressed and MSM with HIV who did not know their status.

**Results:** We enrolled 297 participants of whom 150 received MSM peer HIVST kit distribution (intervention group) and 147 received TASO standard of care HIV testing (control group). Provider cost for the intervention was $2,276 compared with $1,827 for SOC during the 3-month study period. Overall, the intervention resulted in higher HIV positivity yield (4.9 vs. 1.4%) and averted more HIV infections per quarter (0.364 vs. 0.104) compared with SOC. The cost per person tested was higher for the intervention compared to SOC ($15.90 vs. $12.40). Importantly, the cost per new HIV diagnosis ($325 vs. $914) and cost per transmission averted ($6,253 vs. $ 17,567) were lower for the intervention approach relative to SOC. The incremental cost per HIV transmission averted by the self-testing program was $1,727. The incremental cost to providers per additional HIV-positive person identified by the intervention was $147.30.

**Conclusion:** The intervention strategy was cost-effective, and identified more undiagnosed HIV infections than SOC hotspot testing at a cost-effectiveness threshold of US $2,129. Secondary distribution of HIVST kits through peers should further be evaluated with longer duration aimed at diagnosing 95% of all persons with HIV by 2030; the first UNAIDS 95-95-95 target.

## Introduction

Key populations in Uganda, including sex workers, fisher folk, prisoners, and Men having sex with Men (MSM), are disproportionately affected by HIV and account for more than a third of new HIV infections (1, 2). The risk of HIV acquisition is estimated to be 28 times higher among MSM than heterosexual men (3). In 2012, HIV prevalence among MSM (13.2%) (3) was thrice that of heterosexual adult men aged 15–49 years (4.3%) in Uganda (4). A mathematical model suggests that the biggest reductions in HIV incidence in Sub-Saharan Africa will occur through increased coverage of HIV testing and effective treatment of people living with HIV (5). In Uganda, HIV testing uptake among men is low (55%) compared to 82% among women (6). No data are available regarding HIV testing coverage among MSM in Uganda where same sex relationships are criminalized through colonial-era laws (7, 8). The Anti-Homosexuality Act was passed in 2014 but subsequently overturned by the Constitutional Court of Uganda (7, 8). However, social and healthcare stigma and discrimination still hamper key population access to HIV prevention services (6) despite the fact that the Uganda Ministry of Health prohibits discrimination of key populations (7, 8).

Scaling up cost effective strategies for HIV testing and counseling services is paramount in order to effectively reach individuals unaware of their HIV status and/or embedded in risky sexual networks. HIV self-testing (HIVST) is the process by which a person performs an HIV test by themselves to know their HIV sero-status (9). OraQuick is approved by the Ministry of Health, and HIVST is recommended in national guidelines as an additional approach to HIV testing services (9). It is an accessible prevention tool that can empower MSM to overcome stigma and discrimination and increase access to HIV testing. Delivering HIVST through peer and sexual networks (9) could be synergistic to existing MSM HIV prevention programmes by reaching MSM, a high-burden population with limited access to HIV testing services, with user-friendly technology (10–13). Prior studies suggest that most MSM would be willing to distribute HIVST kits as well as to self-test in the presence of a peer or sexual partner (9, 14–18).

An internet peer MSM HIVST kit distribution strategy in the United states averted 3.34 HIV transmissions among 1,325 MSM over 12 months and saved 14.86 QALYs and $1.6 million in lifetime HIV treatment costs with an incremental cost effectiveness ratio (ICER) of $63,400 for cost-effectiveness at $100,000 cost per QALY threshold (10). To our knowledge, no prior study in sub-Saharan Africa has estimated the cost-effectiveness of peer distributed HIV oral fluid self-test kits in MSM sexual and social networks. Understanding cost effectiveness of HIVST kits peer distribution is important to inform programmes and policy makers as HIVST is scaled up. This study aimed to estimate the cost per person tested, incremental cost per HIV infection averted, and cost effectiveness of MSM peer distribution of HIVST kits in Uganda.

## Methods

### Study Design

We conducted a cost effectiveness analysis of a non-randomized study using a provider perspective (9). We compared the cost-effectiveness of an intervention consisting of MSM peer HIVST kit distribution strategy in identifying undiagnosed HIV infection with the standard of care (SOC) HIV testing approach (hotspot HIV testing) used at The AIDS Support Organization (TASO).

### Study Setting

TASO is the largest and oldest indigenous non-governmental HIV care provider in sub-Saharan Africa. It was founded in 1987 by a group of people living with, or deeply affected, by HIV/AIDS in order to provide psychosocial support and basic medical care to people living with HIV and AIDS. TASO Entebbe and Masaka are two of the 11 TASO HIV care centers of excellence located in Central region of Uganda. By June 2018, TASO Entebbe and Masaka had active client populations of >6,000 and >8,000, respectively. The study was conducted in two urban sites, located in Entebbe and Masaka, in Central Uganda.

### Description of HIV Testing Strategies

The TASO SOC HIV testing models included hotspot HIV testing and counseling for key populations, and highly stigmatized persons (9). From January-March 2018, TASO healthcare workers performed hotspot HIV testing fortnightly at MSM hotspots identified in partnership with MSM civil society organizations; MSM were mobilized for HIV testing through social networks, social media and word-of-mouth. They were eligible to receive an HIV test if they were: (i) aged >18 years or older, and (ii) a member of the identified hotspot. For the intervention, we identified 15 MSM peers, eight in Entebbe and seven in Masaka. Each peer (a person with or without HIV and trusted by MSM community) received 10 serialized HIVST kits (Oraquick® Rapid HIV-1/2 Antibody Test, Orasure Technologies, Bethlehem, PA) to distribute to individuals (henceforth referred as participants) in their social and sexual networks who had not tested in the previous 6 months. OraQuick® is a U.S. Food and Drug Administration and Uganda Ministry of Health approved in-home test for HIV-1 and HIV-2 that uses oral fluids. The kit consists of a test swab to collect oral fluid from the user's gums, which is then placed in buffered developer solution and results read after 20–40 min. Peers trained the participants on how to use the HIVST kit and interpret the results. Peers provided pre- and post-test HIV counseling, followed up participants through phone calls and face-to-face meetings, collected used kits, and linked those who tested positive to a blood-based confirmatory HIV testing and ART initiation as previously reported (9) (Table 1). To be eligible for MSM peer HIVST kit distribution, participants: (i) were identified by peers, (ii) aged 18 years or older, (iii) had receptive or insertive anal sex with men in the past year.

**Table 1:** Comparison of MSM peer HIVST kits distribution (intervention) and hotspot HIV testing (standard of care) at the AIDS support organization in Uganda.

**Variable**	**Standard of care hotspot HIV testing**	**Intervention (Peer HIVST distribution)**
Type of test kit	Blood based HIV rapid test kits (Determine® and Uni-Gold®)	Oral fluid based OraQuick® HIV self-test kit
Mobilization of participants	Healthcare workers and drop in center leadership	Peers (MSM either HIV infected or not, identified by MSM community)
Performer	Healthcare worker	Self or peer assisted or HIVST
Where	Hotspots where MSM meet	Place of participant's choice
Linkage to care	Referral letters given by the healthcare workers	Peers used phone calls, face to face meeting including physically linking MSM to confirmatory testing and care
HIV counseling	Healthcare worker	Peers (both HIV-positive and negative MSM)
Sample size	147 participants	150 participants
Completed the test	147 participants	143 participants
Duration	January–March 2018	June–August 2018

### Cost Data Collection and Analysis

We used an ingredients approach to estimate the cost of MSM peer HIVST kit distribution and hotspot SOC approaches (11). For intervention cost estimation, we retrieved and reviewed 2020 intervention expenditures reported in US dollars which included formative research, administrative, overhead costs, and intervention implementation costs from financial records and reports. We extracted research project expenditure including both costs for formative research and peer HIVST kit distribution. Thereafter, we identified MSM peer HIVST kit distribution (intervention) ingredients, estimated costs likely to be provider costs using the expenditure report, and excluded formative research costs. We categorized costs as fixed costs that remained unchanged over the short-term regardless of the number of participants, and variable costs likely to increase or decrease according to the number of participants. Fixed costs included personnel, training, and administration. Variable costs were direct provider costs including HIV test kits, monitoring of peer HIVST kit distribution, and costs of participant tracing and peer stipends. Data collection for the SOC group (January–March 2018) was not synchronous with the intervention group (June–August 2018) (Table 2).

**Table 2:** Estimated costs (US dollars) of the intervention (MSM peer HIVST distribution) and standard of care.

**Programme activities**	**Intervention**	**Standard of care**
	**Peer HIVST distribution provider cost ($)**	**Hotspot HIV testing provider cost ($)**
**Programme start-up costs**		
MSM peer identification and training	148.90	N/A
Venue identification and facilitation	N/A	82.80
**Personnel and Administration cost**		
Project coordinator	449.30	N/A
Counselor coordinator	N/A	720
Laboratory technician	N/A	619.20
Research assistants	168	N/A
Data manager	151	N/A
**Variable costs**		
Transport	250	46
Mobilization of MSM to hotspots	N/A	155
HIV testing	1,008.80	204
Follow up and reporting	99.0	N/A
**Total cost**	**$2,276**	**$1,827**

*The counselor at TASO also works as a Coordinator. The laboratory technician performs HIV testing, manages the data and enters it into the HIV testing register. MSM peers provided pre- and post-test HIV counseling including linkage to a confirmatory test and ART initiation. Transport costs, is for peer transport reimbursement (stipend) for the intervention arm and TASO staff transport to the hotspot for the control arm. HIV testing, is cost for purchasing HIVST kits for the interventional arm and control arm*.

### Standard of Care Costing Estimation

We reviewed TASO key population programme data and reports between January and March 2018 to identify the number of persons tested during hotspot campaigns as previously described (9). Each month, TASO staff conducted a maximum of two hotspot testing sessions. A total of nine hotspot-testing activities were conducted during the study period, five in Entebbe and four in Masaka. We interviewed TASO staff to estimate time (in hours) spent during hotspot testing. We identified ingredient activities to identify provider costs of hotspot testing. We used the 2018 public sector cost of $1.02 to estimate the total cost of Determine® HIV1/2 rapid test kits (Alere Medical Company, Chiba, Japan).

For both groups, we estimated: (a) the cost per person tested by dividing total costs by number of HIV tests completed, (b) the cost per new HIV diagnosis by dividing total costs by the number of new (not diagnosed before) HIV infections identified, and (c) the cost per transmission averted by dividing total programme cost by the number of HIV infections averted.

### Cost-Effectiveness Analysis

Cost effectiveness was defined as the number of HIV transmissions averted using a Bernoulli model to estimate averted transmissions among MSM (12). The number of transmissions averted was estimated using the difference in HIV annual transmission rate between HIV-positive MSM who knew their status and were not virologically suppressed (6.9%) and HIV-positive MSM who did not know their status (12.1%) (12). MSM who do not know their HIV status transmit HIV infection at a higher annual rate than those who know their HIV status (12). Since all MSM who completed an HIV test in both groups received their test results and were initiated on treatment, we assumed that the HIV transmission rate dropped after HIV diagnosis and immediate initiation of treatment. We therefore estimated the number of HIV transmissions averted by multiplying the number of new diagnoses by the difference in HIV transmission rates before and after HIV diagnosis. This was calculated for each HIV testing approach using the formula a = Nu (Tu–Ta) where a is the number of averted HIV transmissions, Nu is undiagnosed HIV infections, Tu is the average HIV transmission rate from MSM unaware of their status, and Ta is the average HIV transmission rate from MSM aware of their status (13).

The cost-effectiveness threshold was set at US $2,130, following the World Health Organization (WHO) “CHOosing Interventions that are Cost-Effective (CHOICE)” recommended threshold for cost effectiveness analysis, i.e., thrice the Uganda gross domestic product per capital of US $710 in 2018 (18–20). We used WHO threshold because we found no comparable HIV prevention (HIV testing) study estimating QALY gained or DALY averted among MSM in a similar setting and did not collect quality of life data. We calculated the incremental cost effectiveness ratio (ICER), defined as ΔC/ΔE = Cb–Ca/Eb–Ea where **C** is total programme cost, **E** is effectiveness (averted infections), **b** is the control index, and **a** is the intervention.

### Sensitivity Analysis

We tested the robustness of the intervention cost effectiveness analysis by using a weighted average transmission rate half (6.9%/2 = 3.45%) the transmission rate for MSM aware of their HIV-positive status and not virologically suppressed (12), taking into consideration participants who completed an HIV test, diagnosed HIV- positive and initiated on ART with good peer adherence support system to achieve viral suppression. For our sensitivity analysis, we assumed that newly diagnosed MSM in the intervention group were more likely to be linked to care by peers and initiate ART than those in the SOC group because the intervention participants received HIV counselling and prevention messaging from a peer who is familiar with them. Thus, we halved the proportion of MSM who engaged in risky behaviours in the intervention group. We also added the cost of confirmatory HIV testing ($1.02 per Determine® rapid test and $3.40 per Uni-Gold® rapid test (2018 market price) for the eight participants diagnosed with HIV infection using HIVST. We also included personnel costs of confirmatory testing, assuming ~30 min of HIV testing and counseling were equivalent to 3.4% effort (mean monthly salary of $800 per TASO provider).

### Ethics Approval

This study was approved by the Infectious Diseases Institute Scientific Review Committee, TASO Research Ethics Committee, The University of California, San Francisco Ethics Committee, and the Uganda National Council for Science and Technology (UNCST). The English or Luganda (local language) information sheet and the verbal informed consent tool approved by the TASO Research Ethics Committee and UNCST was explained to the participant. Those who agreed to take part in the study provided verbal consent that was not documented, consistent with guidelines from regulatory bodies concerned about the criminalization of MSM in Uganda (21).

## Results

Overall, 297 participants were included in the analysis of which 150 received HIVST (intervention) and 147 were reached with SOC HIV testing during the 3-month study period as previously described (9). A total of 143 participants (95%) completed HIVST, of whom 32% had never tested for HIV. All participants in the control group (100%) received SOC testing. Overall, a total of 12 participants were diagnosed with HIV infection: eight in the intervention group and four in the SOC group [5.6 vs. 2.7%, respectively; *P* = 0.02]. All participants newly diagnosed HIV-positive using HIVST received confirmatory HIV testing, were linked to care by the peers and initiated on treatment. Details about SOC and HIVST.

The total provider cost for MSM peer HIVST distribution (intervention) was $2,276 compared with $1,827 for hotspot testing (SOC). Using the HIV transmission rate averted between MSM of known status but not virally suppressed, and unknown HIV status, the intervention resulted in a higher HIV positivity rate (4.9 vs. 1.4%) vs. SOC during 3 months of implementation. Compared to the control group, the intervention strategy averted more HIV transmissions per quarter (0.364 vs. 0.104) but yielded a higher cost per person tested ($15.90 vs. $12.40). The cost per new HIV diagnosis ($914 vs. $325) and the cost per HIV transmission ($17,567 vs. $6,253) averted were higher for the SOC than the intervention. The incremental cost per transmission averted by the self-testing program was $1,727 (Table 3).

**Table 3:** Cost effectiveness of MSM peer HIVST kit distribution and hotspot HIV testing in Uganda.

**Measure**	**MSM peer HIVST kits distribution**	**Hotspot HIV testing**
a. Number of tests^a^	143	147
b. Total number testing positive	8	4
c. Number testing positive, aware	1	2
d. Number testing positive, unaware	7	2
e. Proportion testing positive, unaware	0.049	0.014
f. Transmission rate from MSM unaware HIV+	0.121	0.121
g. Transmission rate from MSM aware HIV+	0.069	0.069
h. Number of infections averted^b^, ^c^	0.364	0.104
i. Total provider costs ($)	2,276	1,827
j. Cost per person tested	15.90	12.40
k. Cost per new diagnosis ($)	325	914
l. Cost per averted infection ($)	6,253	17,567
Incremental CE ratio (ICER), per averted infection	$1,727	

a *Market price of 2018 per Determine HIV test ($1.02) was used to estimate the cost of hotspot testing*.

b The number of averted HIV infections was estimated by multiplying the number of MSM with HIV who became aware of their status and the difference in transmission rates before and after knowing their HIV status. h = d ^*^ [f–g].

c *The average HIV transmission rate for all groups was used for the number of averted infections*.

In sensitivity analysis, adding the cost of confirmatory costing increased the cost per person tested from $15.90 to $16.50 and halving the the transmission rate for MSM aware of their HIV-positive status and not virologically suppressed increased the number of HIV infections averted from 0.364 to 0.602. The cost per infection averted reduced from $6,253 to $3,914. MSM peer HIVST distribution remained cost-effective (ICER $1,062) in identifying new infections and the incremental cost per transmission averted by the self-testing program remained cost effective (Table 4).

**Table 4:** Cost effectiveness of MSM peer HIVST kit distribution and hotspot HIV testing for different HIV transmission rates by type of testing.

**Measure**	**Hotspot**	**MSM peer HIVST kits distribution and testing**	**MSM peer HIVST kits distribution and**
	**HIV testing**		**HIV testing**
**No risky behavior: risky behavior**	**1:1**	**1:1**	**1:1/2**
a. Number of tests	147	143	143
b. Number testing positive, unique	4	8	8
c. Number testing positive, aware	2	1	1
d. Number testing positive, unaware	2	7	7
e. Portion of number testing positive, unaware	0.014	0.049	0.049
f. Transmission rate from unaware HIV+	0.121	0.121	0.121
g. Transmission rate from aware HIV+	0.069	0.069	**0.035**
h. Number of infections averted^a^, ^b^	0.104	0.364	0.602
i. Total provider testing costs^c^ ($)	1,827	2,276	2,356
j. Cost per test completed ($)	12.40	15.90	16.50
k. Cost per new diagnosis ($)	914	325.10	337
l. Cost per averted infection ($)	17,567	6253	3,914

a *The number of averted HIV infections was estimated by multiplying the number of MSM with HIV who became aware of their status and the difference in transmission rates before and after knowing their HIV status. h* = *d* ^*^ *[f–g]*.

b *The average HIV transmission rate for all groups was used for the number of averted transmissions*.

c *We added cost of confirmatory testing using rapid test kit market rate of 2018 and provider cost of counseling and testing*.

The incremental cost to providers per additional HIV-positive person identified by the intervention was $147.30 (Table 5).

**Table 5:** Providers incremental cost for the peer HIVST distribution strategy.

**Strategy**	**Provider costs**	**Incremental cost $**	**Effectiveness (HIV positive diagnosed)**	**Incremental effectiveness (+ve)**	**ICER ($)**
Standard of care arm	1,827	–	4	–	
Intervention arm	2,276	449	8	4	112.3

## Discussion

To our knowledge, this is first study to examine the cost-effectiveness of peer HIVST distribution in sub-Saharan Africa. Our provider perspective analysis found that the MSM peer HIVST kit distribution strategy was cost-effective than SOC hotspot testing in identifying undiagnosed HIV infections among MSM who are a hidden, highly stigmatized population in Uganda and hard to reach with HIV services. The average cost per person tested through MSM peer HIVST kit distribution was higher than hotspot testing because of the higher cost of the Oraquick® HIVST kit relative to the Determine® HIV rapid kit ($6.72 vs. $1.02), respectively. The MSM peer HIVST kits distribution strategy averted thrice as many HIV infections as hotspot testing, potentially lowering the risk of HIV transmission from MSM unaware of their HIV status.

Previous studies have found that HIVST distribution strategies are cost-effective for heterosexual populations in sub Saharan Africa (22, 23). Our findings are in agreement with an internet MSM peer HIVST kits distribution strategy that reached 1,325 MSM over a 12 month period and found that peer based HIVST kit distribution was cost-effective in the United States (10). Furthermore, a modeling study of HIV testing interventions that included lifetime treatment, quality-adjusted life years and 12 months of implementation still found that HIVST delivered through social and sexual networks remained cost effective for identifying undiagnosed HIV infections (17). Reaching high-risk MSM with HIV testing services is key to facilitating early diagnosis of HIV infection and linkage to HIV services. Immediate initiation of antiretroviral therapy has personal health benefits (decreased morbidity and mortality) and public health benefits (prevention of sexual transmission of HIV) (24). MSM peer HIVST kit distribution could address gaps in HIV testing services by increasing testing coverage and frequency and increasing the proportion of first time testers and new HIV diagnoses in stigmatized high-risk populations such as MSM and sex workers in sub-Saharan Africa (15, 25, 26).

The cost per person tested in our study ($15.90 and $12.40 for peer distribution and hotspot testing, respectively) compares favorably with prior studies in sub-Saharan Africa in which the average cost per person tested using door to door community based HIVST distribution was $13.00 (range, $8.78–$16.42): $8.78 in Malawi, $16.42 in Zambia, and $13.84 in Zimbabwe (22). Door-to-door and peer HIVST distribution are community-based HIV testing strategies with similar costs per person tested, but only the latter approach is suitable for hidden populations like MSM. The cost per HIV kit distributed in our peer distribution programme would significantly decrease if it were integrated within the established TASO key population HIV prevention programme in which staff and peers mobilize MSM for HIV testing at hotspots and link them to HIV services. Integration would reduce personnel costs and increase the number of MSM reached with HIVST. In the FHI 360 linkage project (23), MSM peers integrated within the HIV testing programme distributed over 500 kits within 3 months, indicating that peers can efficiently distribute HIVST kits, potentially decreasing the cost per HIVST kit distributed and increasing the yield of persons testing HIV-positive. The incremental provider cost per additional HIV positive person identified by MSM peer HIVST kits distribution was $147.30 (Table 5). This additional cost is considered high given that it is almost thrice Uganda national health expenditure on health per capital – spending of $55 in 2017 (27). However, our current study findings show that the MSM peer HIVST kit distribution approach is cost effective in identifying HIV infection in this stigmatized population.

Scale-up of MSM peer based HIV testing approaches in sub-Saharan Africa in general, and in Uganda in particular, is hampered by criminalization of homosexual behavior and scarcity of evidence on the cost-effectiveness of peer distribution programmes (7, 8). Our results suggest that HIVST can efficiently reach a high-risk marginalized population in need of HIV services. The President's Emergency Plan for AIDS Relief (PEPFAR) is working with the Ministry of Health and HIV implementing partners in Uganda through the local capacity initiative to strengthen the national capacity of key population civil society organizations to address barriers to HIV care, support and prevention services among the MSM community and to understand how best to reach them with HIV services (28). Our results will inform HIV programmes and policy makers on key considerations when scaling up peer distribution of HIVST kits to MSM social and sexual networks. In Uganda, peer distribution of HIVST kits is being scaled up for key populations including MSM and male and female sex workers (29–31). Our findings support the cost-effectiveness of this approach. However, there is need to evaluate the cost-effectiveness of HIVST differentiated delivery models for other key populations and the cost-effectiveness of frequent testing for MSM as recommended by WHO. Quality assurance of HIVST kits is needed since products of unknown quality are available on the unregulated market, with attendant risks of false positive/negative results, and underscoring the need to strengthen consumer protections (25, 32).

Sensitivity analyses including the cost of confirmatory testing and a different set of assumptions for differences in transmission risk found that the cost per person tested increased marginally from $15.90 to $16.50. Importantly, the number of infections averted were increased from 36.4% to 60.2% and the cost per infection averted was halved from $6,253 to $3,914. MSM peer HIVST kit distribution remained cost effective in our study (ICER $1,062) (Table 4).

A strength of our study is the first cost-effectiveness evaluation of peer-distributed HIVST for MSM in sub-Saharan Africa. Our study has limitations. Study duration was only 3 months and mostly reached younger MSM in a setting where older MSM are harder to reach but have higher HIV prevalence. However, younger MSM are at higher risk of HIV acquisition despite lower HIV prevalence; risk of HIV infection increases with age (3). The number of HIV self-tests distributed was relatively small and the comparator was hotspot moonlight testing and not facility-based HIV testing services, thus limiting generalizability of our results. We relied on self-report of prior HIV status and some participants may incorrectly have reported their status. MSM peer HIVST kit distribution (June–August 2018) was not synchronous with programmatic SOC testing (January–March 2018); nevertheless, the 3-month offset did not influence cost estimates. The cost analysis was not specified *a priori* and relied on data from study implementation costs reported to the funder. Initial expenditures included costs of formative research and we may have under- or overestimated implementation costs. We used a provider perspective that excludes patient costs, which are a key barrier to accessing HIV testing services in Uganda. However, in our intervention, patient costs should have been minimal since HIVST kits were distributed to participants at their locations of preference. Finally, we used transmission risk data from the USA; HIV transmission risk among MSM in Uganda is unknown and is likely different (higher or lower) than the United States.

In conclusion, secondary distribution of HIVST kits by MSM peers was cost-effective and identified more undiagnosed HIV infections than SOC approaches. HIVST peer distribution should further be evaluated with longer durations aimed at underserved and hard-to-reach MSM at risk of HIV infection with the goal of expanding testing coverage to 95% of all persons with HIV (UNAIDS first 95 target).

## Data Availability Statement

The original contributions presented in the study are included in the article/supplementary material, further inquiries can be directed to the corresponding author/s.

## Ethics Statement

The studies involving human participants were reviewed and approved by this study was approved The Infectious Diseases Institute Scientific Review Committee, TASO Research Ethics Committee, The University of California, San Francisco Ethics Committee, and the Uganda National Council for Science and Technology (UNCST). The English or Luganda (local language) information sheet and the verbal informed consent tool approved by the TASO Research Ethics Committee and UNCST was explained to the participant. Those who agreed to take part in the study provided verbal consent that was not documented, consistent with guidelines from regulatory bodies concerned about the criminalization of MSM in Uganda. Written informed consent for participation was not required for this study in accordance with the national legislation and the institutional requirements.

## Author Contributions

SO designed the study and wrote the first draft along with AM. SO, YK, PO, and BC performed the statistical analyses. All authors contributed to data collection, interpretation of the results and the writing of the manuscript, and approved the final draft.

## Conflict of Interest

The authors declare that the research was conducted in the absence of any commercial or financial relationships that could be construed as a potential conflict of interest.

## References

[1] Hladik W Barker J Ssenkusu JM Opio A Tappero JW Hakim A. HIV infection among men who have sex with men in kampala, uganda-a respondent driven sampling survey. PLoS ONE. (2012) 7:e38143. 10.1371/journal.pone.0038143 22693590 PMC3364961

[2] Hladik W Sande E Berry M Ganafa S Kiyingi H Kusiima J. Men who have sex with men in Kampala, Uganda: results from a bio-behavioral respondent driven sampling survey. AIDS Behav. (2017) 21:1478–90. 10.1007/s10461-016-1535-2 27600752

[3] UNAIDS (Joint United Nations Programme on HIV/AIDS). Global AIDS Update (2018) Miles To Go: Closing Gaps Breaking Barriers Righting Injustices. Unaids. Available online at: https://www.unaids.org/sites/default/files/media_as.

[4] Uganda Population Based HIV Impact Assessment 2016-2017 Report.

[5] Kelly SL Wilson DP. HIV cascade monitoring and simple modeling reveal potential for reductions in HIV incidence. J Acquir Immune Defic Syndr. (2015) 69:257–63. 10.1097/QAI.0000000000000655 25886932

[6] King R Sebyala Z Ogwal M Aluzimbi G Apondi R Reynolds S. How men who have sex with men experience HIV health services in Kampala, Uganda. BMJ Global Healt. (2020) 5:e001901. 10.1136/bmjgh-2019-00190129376472

[7] Uganda Penal Code Act Chapter 120 Section 145 Laws of Uganda. (1950). Available online at: https://ulii.org/ug/legislation/consolidated-act/120 (accessed December 4th, 2020).

[8] Oloka-Onyango & 9 Others vs. Attorney General. Constitutional Petition no. 08 of 2014. Available online at: http://www.ulii.org/ug/judgment/constitutional-court/2014/14/ (accessed May 11, 2020).

[9] Okoboi S Lazarus O Castelnuovo B Nanfuka M Kambugu A Mujugira A. Peer distribution of HIV self-test kits to men who have sex with men to identify undiagnosed HIV infection in Uganda: a pilot study. PLoS ONE. (2020) 15:e0227741. 10.1371/journal.pone.0227741 31971991 PMC6977761

[10] Shrestha RK Chavez PR Noble M Sansom SL Sullivan PS Mermin JH. Estimating the costs and cost-effectiveness of HIV self-testing among men who have sex with men, United States. J Int AIDS Soc. (2020) 23:e25445. 10.1002/jia2.25445 31960580 PMC6970935

[11] Barton P Bryan S Robinson S. Modelling in the economic evaluation of health care: Selecting the appropriate approach. J Health Serv Res Policy. (2004) 9:110–8. 10.1258/135581904322987535 15099459

[12] Lin F Farnham PG Shrestha RK Mermin J Sansom SL. Cost effectiveness of HIV prevention interventions in the US. Am J Prev Med. (2016) 50:699–708. 10.1016/j.amepre.2016.01.011 26947213

[13] Castel AD Choi S Dor A Skillicorn J Peterson J Rocha N. Comparing cost-effectiveness of HIV testing strategies: targeted and routine testing in Washington, DC. PLoS ONE. (2015) 10:1–11. 10.1371/journal.pone.0139605 26465771 PMC4605630

[14] Sharma A Chavez PR MacGowan RJ McNaghten AD Mustanski B Gravens L. Willingness to distribute free rapid home HIV test kits and to test with social or sexual network associates among men who have sex with men in the United States. AIDS Care. (2017) 29:1499–503. 10.1080/09540121.2017.1313386 28393612 PMC12186660

[15] Lippman SA Lane T Rabede O Gilmore H Chen YH Mlotshwa N. High acceptability and increased HIV-testing frequency after introduction of HIV self-testing and network distribution among South African MSM. J Acquir Immune Defic Syndr. (2018) 77:279–87. 10.1097/QAI.0000000000001601 29210826 PMC5807184

[16] Lippman SA Lane T Rabede O Gilmore H Chen YH Mlotshwa N. HIV self-test distribution increases test frequency in South African MSM. Top Antivir Med. (2018) 26:63s−64s.

[17] Hutchinson AB Farnham PG Sansom SL Yaylali E Mermin JH. Cost-effectiveness of frequent HIV testing of high-risk populations in the United States. J Acquir Immune Defic Syndr. (2016) 73:323–30. 10.1097/QAI.0000000000000838 26361172 PMC4770372

[18] Woods B Revill P Sculpher M Claxton K. Country-level cost-effectiveness thresholds: initial estimates and the need for further research. Value Health. (2016) 19:929–35. 10.1016/j.jval.2016.02.017 27987642 PMC5193154

[19] Marseille E Larson B Kazi DS Kahn JG Rosen S. Thresholds for the cost–effectiveness of interventions: alternative approaches. Bull World Health Organ. (2015) 93:118–24. 10.2471/BLT.14.138206 25883405 PMC4339959

[20] Uganda GDP Growth (annual). Budgeting and Public Expenditures in OECD Countries. Kampala: Uganda National Council for Science and Technology (2019).

[21] Uganda National Council for Science & Technology. National Guidelines for Research Involving Humans as Research participants. (2014).

[22] Mangenah C Mwenge L Sande L Ahmed N d'Elbée M Chiwawa P. Economic cost analysis of door-to-door community-based distribution of HIV self-test kits in Malawi, Zambia, and Zimbabwe. J Int AIDS Soc. (2019) 22:e25255. 10.1002/jia2.25255 30907499 PMC6432106

[23] FHI360 Linkage Project. (2020). Available online at:https://www.fhi360.org/sites/default/files/media/documents/resource-linkages-burundi-achievements.pdf. (accessed April 20, 2020).

[24] Cohen MS Chen YQ McCauley M Gamble T Hosseinipour MC Kumarasamy N. Antiretroviral therapy for the prevention of HIV-1 transmission. N Engl J Med. (2016) 375:830–9. 10.1056/NEJMoa1600693 27424812 PMC5049503

[25] Thirumurthy H Masters SH Mavedzenge SN Maman S Omanga E Agot K. Promoting male partner HIV testing and safer sexual decision making through secondary distribution of self-tests by HIV-negative female sex workers and women receiving antenatal and post-partum care in Kenya: a cohort study. Lancet HIV. (2016) 3:e266–74. 10.1016/S2352-3018(16)00041-2 27240789 PMC5488644

[26] Sharma M Ying R Tarr G Barnabas R. Systematic review and meta-analysis of community and facility-based HIV testing to address linkage to care gaps in sub-Saharan Africa. Nature. (2015) 528:S77–85. 10.1038/nature16044 26633769 PMC4778960

[27] Uganda National Health Expenditure 2017 Report.

[28] Fact Sheet. PEPFAR Strategy for Accelerating HIV/AIDS Epidemic Control (2017-2020). Kampala: PEPFAR (2017). p. 18.

[29] Uganda Ministry of Health. Distribution of HIV Self Test Kits to Public Health Facilities. Geneva: WHO (2018).

[30] WHO Policy Brief. Consolidated Guidelines on HIV Testing Services for a Changing Epidemic. Geneva: WHO (2019).

[31] WHO Policy Brief. Who recommends social network-based HIV testing approaches for key populations as part of partner services package. JAMA. (2014) 312:1–8.

[32] MacPherson P Lalloo DG Webb EL Maheswaran H Choko AT Makombe SD. Effect of optional home initiation of HIV care following HIV self-testing on antiretroviral therapy initiation among adults in Malawi: a randomized clinical trial. JAMA. (2014) 312:372–9. 10.1001/jama.2014.6493 25038356 PMC4118051

